# Mechanical Purification of Lipofilling: The Relationship Between Cell Yield, Cell Growth, and Fat Volume Maintenance

**DOI:** 10.1007/s00266-024-03870-0

**Published:** 2024-03-20

**Authors:** Pietro Gentile, Riccardo Ossanna, Lindsey Alejandra Quintero Sierra, Andrea Sbarbati

**Affiliations:** 1 Department of Surgical Science, Tor Vergata” University, Via Montpellier 1, 0017300133 Rome, Italy; 2 Academy of International Regenerative Medicine & Surgery Societies (AIRMESS), 1201 Geneva, Switzerland; 3 Department of Neuroscience, Biomedicine, and Movement Sciences, University of Verona, 37124 Verona, Italy

**Keywords:** Adipose-derived stromal vascular fraction cells, Stromal vascular fraction, AD-SVFs, Adipose-derived mesenchymal stem cells, ADSCs, Centrifugation, Filtration, enzymatic digestion, Regenerative plastic surgery, plastic surgery

## Abstract

**Background:**

The mechanical manipulations of fat tissue represented from centrifugation, filtration, washing, and fragmentation were considered the most effective strategies aiming to obtain purified lipofilling with different impacts both in terms of adipose-derived stem cells amount contained in stromal vascular fraction, and fat volume maintenance.

**Objectives:**

The present work aimed to report results in fat volume maintenance obtained by lipofilling purification based on the combined use of washing and filtration, in a clinical study, and to deeply investigate the adipose-derived stem cells yield and growth capacity of the different stromal vascular fraction extraction techniques with an in vitro approach.

**Methods:**

A preliminary prospective, case-control study was conducted. 20 patients affected by face and breast soft tissue defects were treated with lipofilling and divided into two groups: *n* = 10 patients (study group) were treated with lipofilling obtained by washing and filtration procedures, while *n* = 10 (control group) were treated with lipofilling obtained by centrifugation according to the Coleman technique. 6 months after the lipofilling, the volume maintenance percentage was analyzed by clinical picture and magnetic resonance imaging comparisons. Additionally, extracted stromal vascular fraction cells were also in vitro analyzed in terms of adipose-derived stem cell yield and growth capacity.

**Results:**

A 69% ± 5.0% maintenance of fat volume after 6 months was observed in the study group, compared with 44% ± 5.5% in the control group. Moreover, the cellular yield of the control group resulted in 267,000 ± 94,107 adipose-derived stem cells/mL, while the study group resulted in 528,895 ± 115,853 adipose-derived stem cells /mL, with a *p*-value = 0.1805. Interestingly, the study group showed a fold increase in cell growth of 6758 ± 0.7122, while the control group resulted in 3888 ± 0.3078, with a *p* < 0.05 (*p* = 0.0122).

**Conclusions:**

The comparison of both groups indicated that washing and filtration were a better efficient system in lipofilling preparation, compared to centrifugation, both in terms of volume maintenance and adipose-derived stem cell growth ability.

**Level of Evidence III:**

This journal requires that authors assign a level of evidence to each article. For a full description of these Evidence-Based Medicine ratings, please refer to the Table of Contents or the online Instructions to Authors http://www.springer.com/00266.

**Supplementary Information:**

The online version contains supplementary material available at 10.1007/s00266-024-03870-0.

## Introduction

Soft tissue defects in plastic surgery and several pathologies like as hemifacial atrophy [1], Romberg syndrome [2], ulcers [3, 4], outcomes of breast cancer [5, 6], breast hypoplasia and tuberous breast [7, 8], scars [9], and face soft tissue effects [10] have been treated with greater benefits thanks to the lipofilling. Many strategies for autologous lipofilling preparation have been reported aiming to improve long-term fat volume maintenance. Currently, several discussions seem to relate fat maintenance directly with the number of adipose-derived stem cells (ADSCs) contained in the stromal vascular fraction (SVF) of fat tissue. SVF is composed of heterogeneous cells with progenitor activity including pre-adipocytes, ADSCs, pericytes, endothelial cells, and macrophages thus providing immunomodulatory capacity for lipofilling [11]. For these reasons, several procedures of adult SVF cells (SVFs) isolation, using minimal and substantial manipulation methods, have been described [6–11]. The European Medicines Agency (EMA), and the US Food and Drug Administration (FDA), described adult cells as biological products obtained from two different methods of manipulation: “Minimal Manipulation” and “Substantial Manipulation.” Minimal manipulation does not modify the biological characteristics and functions of cells. It is based on filtration, centrifugation, cutting decantation, fragmentation, cryopreservation, grinding, vitrification, shaping, freezing, lyophilization, soaking in antibiotics, cell separation/concentration/purification, irradiation, sterilization, without any cell expansion [6–12]. Substantial manipulation is based on procedures of cell expansions and related cultures, cell differentiation, cell activation, genetic modification, and any processing that alters the biological, physiological, or structural characteristics of cells or tissues [11]. Minimal and substantial manipulation procedures have been detailed and described in the scientific literature, sometimes with conflicting results, presenting many different techniques that tried to find the best method of isolating SVFs and fat purification [6–12]. Minimal manipulation procedures, based on centrifugation, filtration, washing, and fragmentation of the fat tissue, appeared as the most frequent strategies adopted to purify fat graft. In the last years, SVFs isolation procedures and lipofilling purification methods have deeply shifted from enzymatic digestion to mechanical processing [6–12]. Enzymatic digestion of a tissue to release cells is considered to be substantial manipulation, when the aim is to dissociate cell-cell contacts, and the released cells are administered into patients with or without subsequent manipulation [13]. If the number of certain cells (e.g., mesenchymal stem cells—MSCs—in fat grafts) is enriched by selection and the processing does not change the characteristics of the cells, this is not considered a substantial manipulation [14]. Of the mechanical procedures, the washing seems to be that with less clinical data in terms of correlation between the SVFs amount, ADSCs number, and the fat volume maintenance [6–12].

This article aimed to report the preliminary clinical results, represented by fat volume maintenance and ADSCs yield and growth, obtained by filtration and washing procedures of fat tissue.

## Methods

### Study Protocol

A prospective case-control study that is categorized as evidence-based medicine (EBM) level 3 was carried out in strict accordance with the Declaration of Helsinki and with internationally consented ethics in clinical research [15]. A quality evaluation was performed using the STROBE (Strengthening the Reporting of Observational Studies in Epidemiology) checklist [16]. The protocols were fully adopted in accordance with the European Medicines Agency (EMA) and Committee for Advanced Therapy (CAT) recommendations (June 20, 2014, EMA/CAT/600280/2010, Rev. 1) and European regulations (EC 1394/2007).

The study was the object of a research contract between the author (P.G.) and the University of Rome “Tor Vergata,” approved by Rectoral Decree R.D. n. #1467/2017, continued in associate professor contract #13489/2021. Before receiving any surgical care, each patient completed an informed consent form that included full disclosure of the study's risks, rewards, and alternative treatment options.

### Patients

**Fig. 1:**
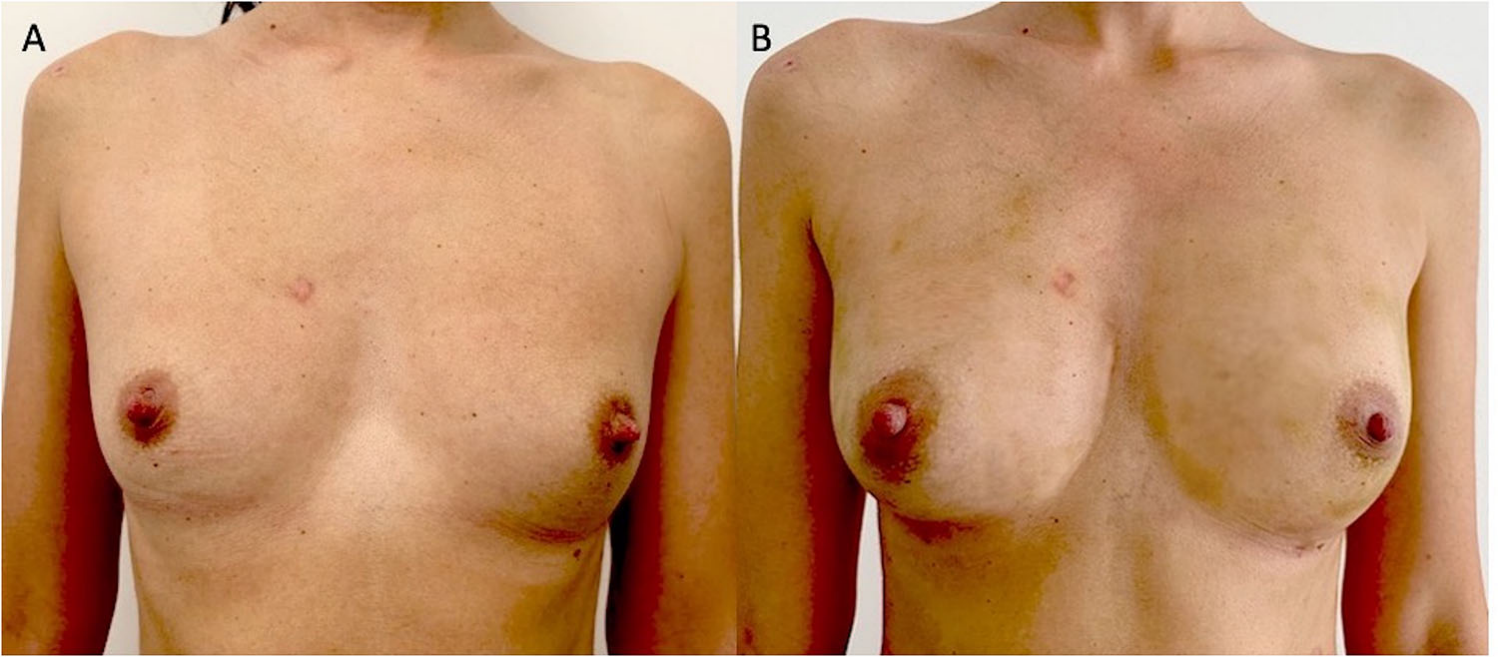
Analysis of SG patient, a 32-year-old female patient affected by bilateral breast hypoplasia. **A** Frontal pre-operative view showing breast hypoplasia of moderate degree; **B** Post-operative in frontal view 6 months (T6) after the first fat graft injection. A great volume improving evenly distributed in the superior and inferior lateral and internal quadrant, peri-areolar region, and inferior and superior pole, obtaining excellent results

**Fig. 2:**
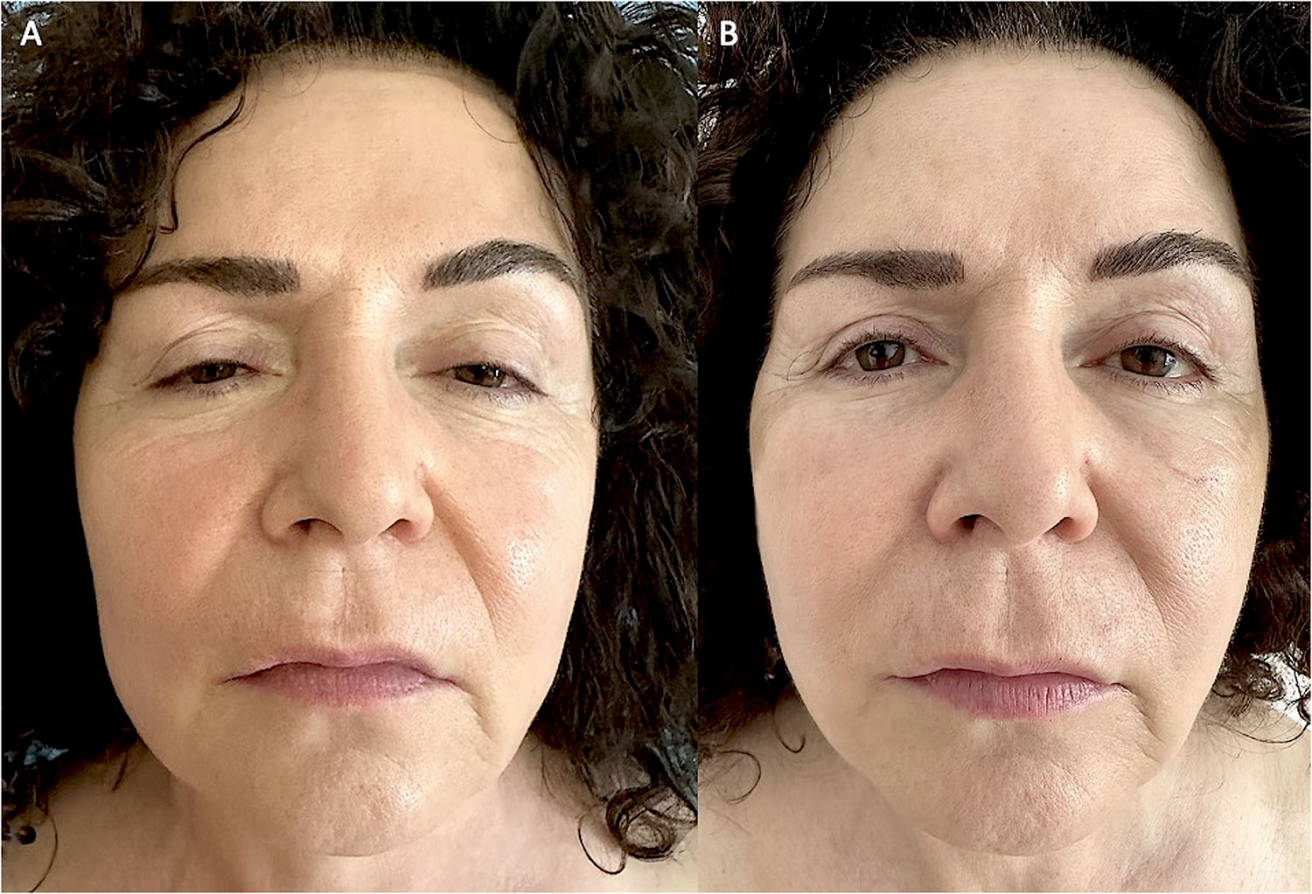
Analysis of SG patient, a 57-year-old female patient affected by signs of aging and loss of elasticity in the face. **A** Frontal pre-operative view showing signs of aging; **B** Post-operative in frontal view 6 months (T6) after the first fat graft injection. A great volume improvement was evenly distributed in the zygomatic, periorbital, and cheek areas, obtaining good results

20 patients affected by face and breast soft tissue defects were treated with lipofilling and divided into two groups:

10 patients (8 females [F] and 2 males [M]) aged 19–61 years (average age 40) affected by breast soft tissue defects (outcomes of breast reconstruction *n* = 1; Tuberous breast *n* = 1; Poland Syndrome *n* = 1; outcomes of scars *n* = 1; breast hypoplasia *n* = 2) (Fig. 1A) and face soft tissue defects (outcomes of scars *n* = 2 [1-F and 1-M]; Romberg Syndrome *n* = 1 [1-M]; signs of aging n = 1) (Fig. 2A) [1-F] were treated with lipofilling obtained by only washing and filtration procedures (study group [SG]).

**Fig. 3:**
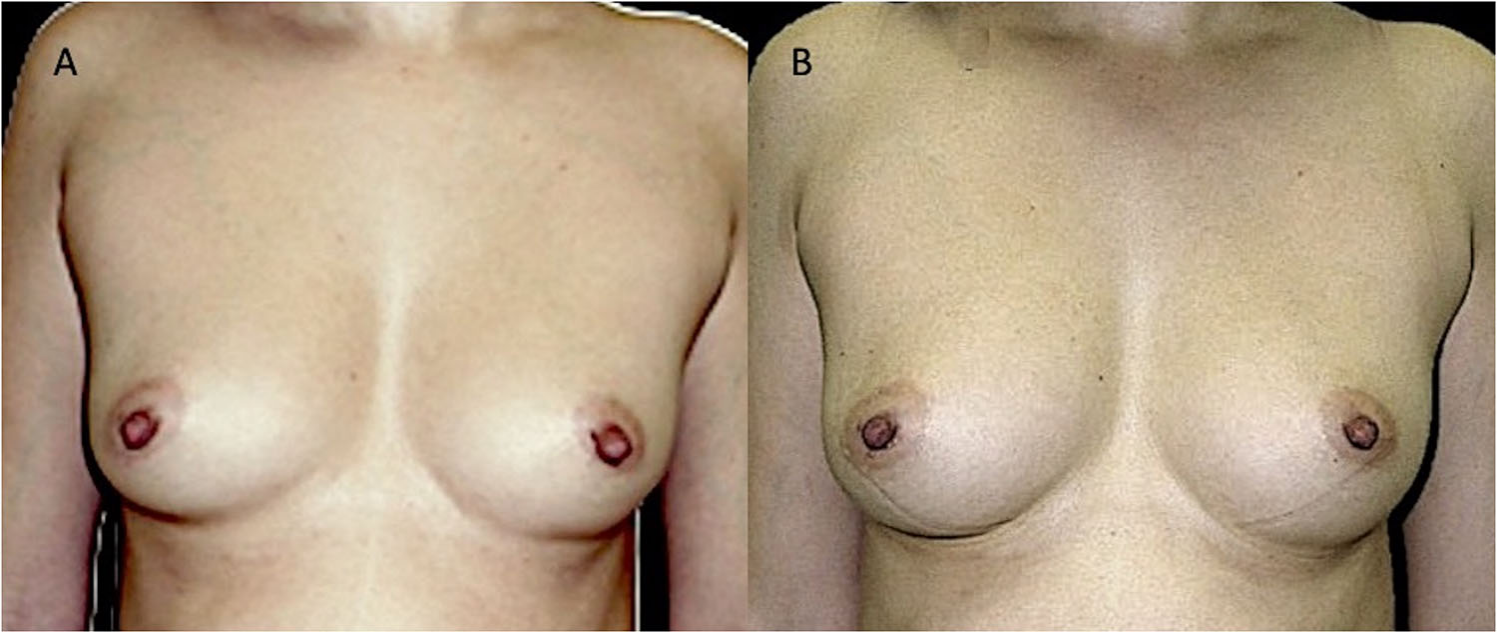
Analysis of CG patient, a 39-year-old female patient affected by bilateral breast hypoplasia. **A** Frontal pre-operative view showing breast hypoplasia of moderate degree; **B** Post-operative in frontal view 6 months (T6) after the first fat graft injection. A enough volume improving evenly distributed in the superior and inferior lateral and internal quadrant, peri-areolar region, and inferior and superior pole, good results

**Fig. 4:**
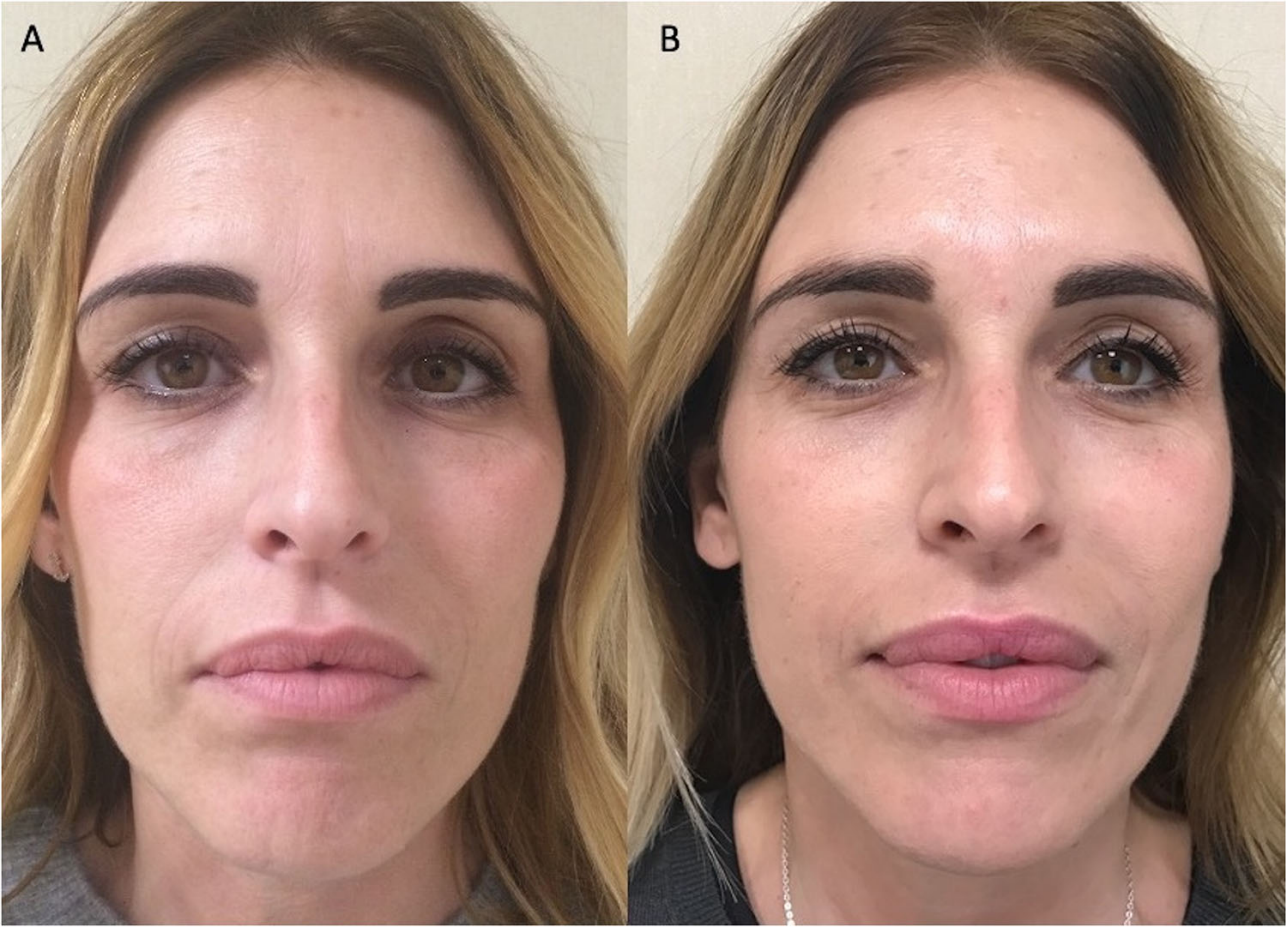
Analysis of CG patient, a 40-year-old female patient affected by signs of aging and loss of elasticity in the face. **A** Frontal pre-operative view showing signs of aging; **B** Post-operative in frontal view 6 months (T6) after the first fat graft injection. A volume improvement was evenly distributed in the zygomatic, periorbital, nasolabial fold, and cheek areas, obtaining enough results

10 patients (8 females [F] and 2 males [M]) aged 18–60 years (average age 39), affected by breast soft tissue defects (outcomes of breast reconstruction *n* = 2; Tuberous breast *n* = 1; outcomes of scars *n* = 1; breast hypoplasia *n* = 2) (Fig. 3A) and face soft tissue defects (outcomes of scars *n* = 2 [1-F and 1-M]; Romberg Syndrome *n* = 1 [1-M]; signs of aging *n* = 1) (Fig. 4A) [1-F] were treated with lipofilling obtained by only centrifugation according to the Coleman technique (control group [CG]).

**Scheme 1:**
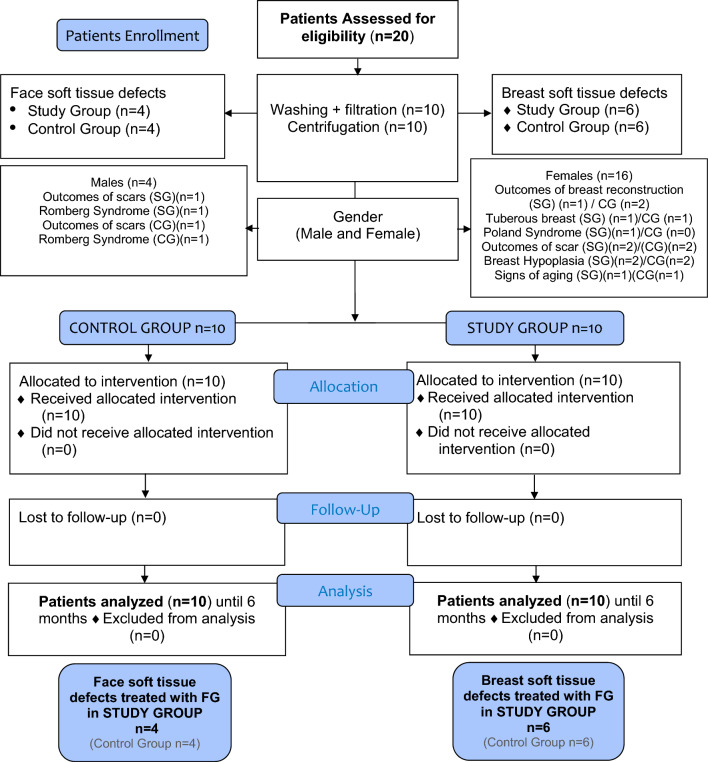
CONSORT (Consolidated Standards of Reporting Trials) flow diagram on patients’ enrollment, including soft tissue defects and treatment assessment

**Fig. 5:**
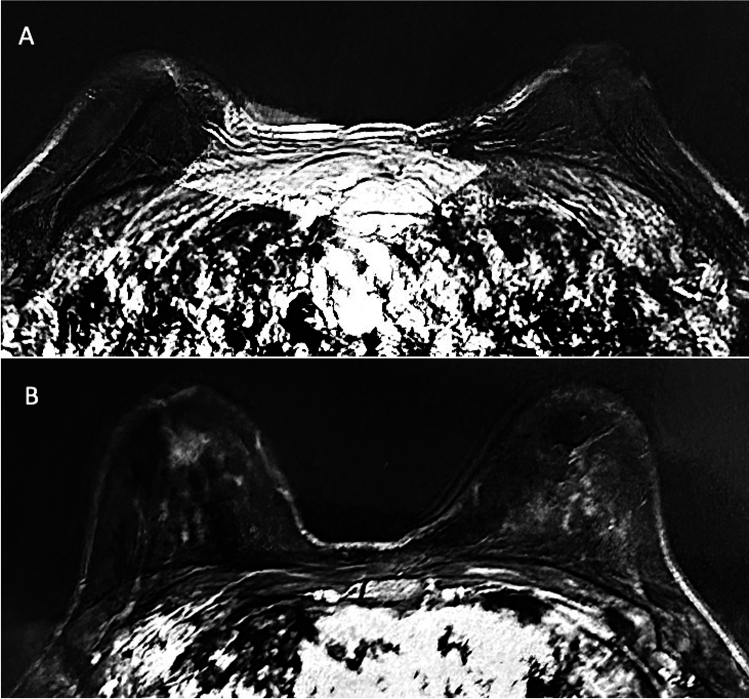
T2-weighted MRI scans of the SG patient showed in Fig. 1. **A** Pre-operative situation of the patient with a high degree of breast hypoplasia bilaterally. **B** Post-operative obtained 6 months later the second fat graft injection with significative volume improvement in the breasts bilaterally

**Fig. 6:**
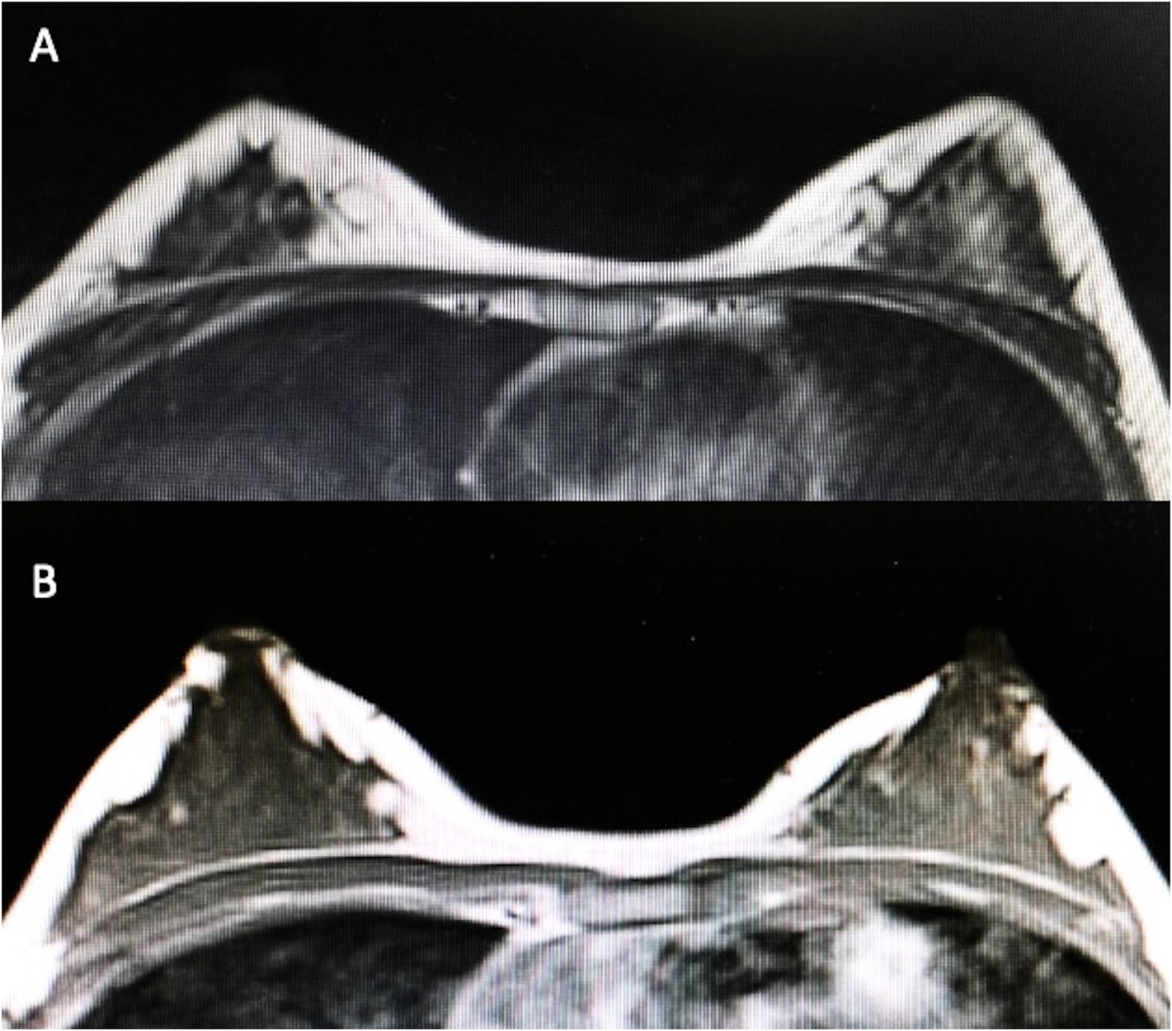
T2-weighted MRI scans of the CG patient showed in Fig. 3. **A** Pre-operative situation of the patient with a high degree of breast hypoplasia bilaterally. **B** Post-operative obtained 6 months later the second fat graft injection with volume improvement in the breasts bilaterally

Patients’ data and clinical assessment were detailed in a CONSORT flow diagram (Scheme 1) and Table 1. All patients recruited have undergone an accurate pre-operative screening based on a thorough clinical assessment, a photographic and instrumental examination using magnetic resonance imaging (MRI) (Figs. 5A, 6A), and ultrasound (US).

**Table 1:** Patient data assessments

	**SG**	**CG**
Number of patients	10	10
Sex	8 F and 2 M	8 F and 2 M
Age	19 - 61 years (40,0)	18 - 60 years (39,0)
Race	Caucasian	Caucasian
BMI at surgery, kg/m2	26,5 (min 18, max 35)	26,5 (min 18, max 35)
Diabetes	0	0
Smoke	2 (20%)	3 (30%)
Pre-menopausal	4 F (50%)	4 F (50%)
Breast soft tissue defects		
(Bilateral Breast BB Unilateral Breast UB)	Outcomes of breast reconstruction (*n* = 1) (BB), Tuberous Breast (*n* = 1) (BB), Poland Syndrome (*n* = 1) (UB), Outcomes of scars (*n* = 1) (UB), Breast hypoplasia (*n* = 2) (BB)	Outcomes of breast reconstruction (*n* = 2) (BB), Tuberous Breast (*n* = 1) (BB), Outcomes of scars (*n* = 1) (UB), Breast hypoplasia (*n* = 2) (BB)
Face soft tissue defects	Outcomes of scars (*n* = 2) [1-F and 1-M] (Lips * n* = 1, Nose * n* = 1), Romberg Syndrome (*n* = 1) [1-M] (Unilateral Zygomatic and cheek area * n* = 1), Signs of aging (*n* = 1) [1-F] (Bilateral Zygomatic, cheek and nasolabial fold area * n* = 1)	Outcomes of scars (*n* = 2) [1-F and 1-M] (Lips * n* = 1, Nose * n* = 1), Romberg Syndrome (*n* = 1) [1-M] (Unilateral Zygomatic and cheek area * n* = 1), Signs of aging (*n* = 1) [1-F] (Bilateral Zygomatic, cheek, and nasolabial fold area * n* = 1)
Only one treatment	8 cases (80%)	7 cases (70%)
Mean transfer volume for each treatment in face soft tissue defects	12mL (range 4–20mL)	12mL (range 4–20mL)
Mean transfer volume for each treatment in breast tissue defects	270mL (range 150–390mL)	270mL (range 150–390mL)
Volume maintenance percentage after one treatment (average)	76% ± 5.0% at T3 (All patients)	52% ± 5.5% at T3 (All patients)
	69% ± 5.0% at T6 (All patients)	44% ± 5.5% at T6 (All patients)
Volume maintenance percentage after one treatment (Breast soft tissue defects)	70% ± 5.0% at T3 (All patients)	46% ± 5.0% at T3 (All patients)
	61% ± 5.0% at T6 (All patients)	36% ± 5.0% at T6 (All patients)
Volume maintenance percentage after one treatment (Face soft tissue defects)	82% ± 5.0% at T3 (All patients)	58% ± 5.0% at T3 (All patients)
	77% ± 5.0% at T6 (All patients)	52% ± 5.0% at T6 (All patients)
Second treatment	2 cases (20%) at T6	3 cases (30%) at T6
	Insufficient final volume re-treated	Insufficient final volume re-treated
Patients excluded	**0**	0
Skin necrosis	0	0
Cyst Formation and calcification	55% (US) T3 / 46% (US) T6	55% (US) T3 / 46% (US) T6
	4,8% (MRI) T3 / 3,2% (MRI) T6	4,8% (MRI) T3 / 3,2% (MRI) T6
Cyto±steatonecrosis	5,5% (MRI) T3-T6	5,5% (MRI) T3-T6
	5,5% (US) T3-T6	5,5% (US) T3-T6

### Inclusion and Exclusion Criteria

Ages 18 to 61, a history of breast, face, and body soft tissue abnormalities, and a BMI between 18 and 35 kg/m2 were all taken into consideration as inclusion criteria. Additional requirements for inclusion in SG and CG were having enough fat tissue at the harvest sites, which included the inner knees, thighs, flanks, and belly. At the same time, exclusion criteria were considered and divided into systemic, local, and psychological. Systemic exclusion criteria were bone marrow aplasia, anti-aggregating therapy, sepsis, cancer, and uncompensated diabetes, while local exclusion criteria were cancer loss of substance and uncontrolled comorbidities. Genetic problems or tobacco smoking were not thought to be barring factors. Psychological diseases such as the presence of body image perception disorder, dysmorphophobia, personality, and/or mood disorders were all considered exclusion criteria.

### Mechanical Procedures

The washing and filtration procedures (SG) were performed using two different systems, respectively, Beauty-Stem™ and Beauty-Stem Duo™ (BPB Medica^®^ - BIOPSYBELL S.R.L. Via Aldo Manuzio 24, 41037 MIRANDOLA (MO), Italy, https://www.biopsybell.com/products/aesthetic/). In both cases, Beauty-Stem™ (Supplemental Figure 1A) and Beauty-Stem Duo™ (Supplemental Figure 1B) (BPB Medica^®^) up to 400ml of fat tissue were collected and purified directly without any procedure of centrifugation, using a continue saline washing procedure, permitting filtering fat tissue through two filters: the first filter has micro-fragmented the adipose tissue retaining the fibrotic tissue while the second filter with a denser mesh retained the micro-fragmented adipose tissue and discharged the pro-inflammatory contents and liquids in the waste bag. The complete washing and filtration of the tissue has been done by continuing to gently move the processing spatula on the filter (Supplemental Figure 1C, D). At the end of the purification, an average of 200 mL of purified fat (100mL/300mL), depending on the device used (duo or mono), ready to be injected, was obtained. The only centrifugation procedure (CG) was performed using the Coleman procedure [17].

### Lipofilling Infiltration

Both groups (SG and CG) received lipofilling injections using the "Gentle technique." [18, 19]

In detail, the author implanted linear deposits of purified fat through slow and controlled movements into the subcutaneous tissue both in case of face and breast soft tissue defects. In the case of breast soft tissue defects correction, the implanting was supra-fascial, retro-glandular, and intra-glandular (but never into the pectoralis major muscle) [12], through multiple tunnels and several different entrances (infra-mammary fold [located at 130°, 180°, and 220°], higher external quadrant [290°], lower-external quadrant [240°], higher internal quadrant [65°] and lower-internal quadrant [110°]) using 1.5mm cannulas, as already described. [12, 19]. Additionally, four different accesses were adopted in the areolar area located at 0°, 90°, 180°, and 270°.

### Clinical and Instrumental Lipofilling Maintenance

Clinical evaluation and quality checks were carried out through picture comparison and defects analysis (site of the defects [breast, face, or body], the degree of the defects [high, moderate, low], presence of scar). Lipofilling volume maintenance evaluation was carried out using MRI and US. Patients were analyzed at 1 week (T1), 2 weeks (T2), 4 weeks (T3), 8 weeks (T4), 12 weeks (T5), and 24 weeks (T6), by clinical examination while at 1 month (T3), and 6 months (T6), later the last procedure by MRI and US. Volumetric fat maintenance was calculated at 6 months (T6) by analyzing MRI scans gathered in axial and sagittal planes and their 3-D reconstructions (ADW 4.0; GE Medical Systems, Milwaukee, Wis.). All examinations were performed and analyzed in a blinded fashion by two radiologists experienced in interpreting MRI imaging.

### Histological Analysis of Fat Tissue Samples

After being fixed with paraformaldehyde 4% (Boster Biological technology co., Ltd) for 20 minutes, adipose tissue samples derived from SG and CG were washed with PBS 1X, dried, and embedded with Optimal Cutting Temperature compound (OCT). Samples were cryosectioned in 14 µm-thick transversal slices. Slides were dried under the hood flow for 30 minutes and then stored at − 20°C for the subsequent histological analysis. To evaluate the morphology of the SG and CG fat tissue samples, slides were rehydrated with distilled water for 2 minutes, stained with hematoxylin (Sigma-Aldrich, Milan, Italy) for 40 seconds, gently washed with running water, stained with 1/10 Eosin (Sigma-Aldrich, Milan, Italy) for 10 seconds, and finally washed with distilled water. Slides were then dehydrated with increasing alcohol concentration (80% for 5 minutes, 95% for 5 minutes, 100% for 5 minutes two times, and xylene for 5 minutes two times). Finally, it was added a drop of mounting medium (Entellan) and the slides were covered with the cover slice. All slides were examined under an Olympus BX-51 microscope (Olympus, Tokyo, Japan) equipped with a digital camera (DKY-F58 CCD JVC, Yokohama, Japan).

### Fat Tissue Sample Digestion

The fat tissue samples (10 mL each) derived from both SG and CG were incubated with collagenase type I at a concentration of 1 mg/mL (GIBCO Life Technology, Monza, Italy) dissolved in Hank’s Balanced Salt Solution (HBSS, GIBCO Life Technology, Monza, Italy) with 2% of Bovine Serum Albumin (BSA, GIBCO Life Technology, Monza, Italy) for 45 min at 37ºC. Complete culture medium (Dulbecco’s Modified Eagle’s Medium (DMEM), Sigma-Aldrich, Italy), supplemented with 10% of Fetal Bovine Serum (FBS, GIBCO Life Technologies, USA), 1% of 1:1 penicillin/streptomycin (P/S solution, GIBCO Life Technologies, USA), and 0.6% of Amphotericin B (GIBCO Life Technologies, USA), was added to neutralize the enzyme action. After the neutralization process, the sample was centrifuged at 3000 rpm for 5 min. The cell pellet was incubated with 1 mL of erythrocyte lysis buffer 1X (Macs Miltenyi Biotec, Milan, Italy) for 10 min at room temperature. Again, the cell suspension was centrifuged and resuspended with 1 mL of complete culture medium. Finally, the cells were filtered through a 70 µm nylon mesh to be seeded in a T25 flask. The extracted cells were evaluated in terms of cellular yield and cell growth.

### Cellular Yield

The extracted ADSCs were counted for cellular yield calculation considering the number of extracted free cells divided by the processed volume of fat. The number of living cells was calculated using the Trypan Blue exclusion assay in a CytoSMART counter (Automated Image-Based Cell Counter, version 1.5.0.16380, CytoSMART Technologies B.V., Eindhoven, Netherlands).

### Cellular Growth Capacity

The extracted ADSCs were seeded on a 25 cm^2^ T-flask with a complete culture medium and incubated in a humidified atmosphere at 37°C with 5% CO_2_. The first medium change was performed after 72h from the enzymatic digestion and the subsequent changes every 48 h. The cellular growth was performed by counting the cells after 7, 14, and 21 days of culture, and the obtained cell numbers were normalized by dividing them with the corresponding cellular yield.

### Statistical Analysis

Statistical analyses were performed using GraphPad Prism 7.03 for Windows (GraphPad Software, La Jolla, CA, USA). For statistical analysis, a 2-way ANOVA test and a t-student test were performed, and a 95,00% confidence interval was employed to compare the evaluated groups considering a *p*-value < 0.05 to indicate the differences were statistically significant.

## Results

### Clinical and Fat Volume Maintenance Outcomes

Regarding the clinical results (Figs. 1, 2, 3, 4B), after the lipofilling, the volume maintenance rates were not similar in SG and CG. A 69% ± 5.0% maintenance of fat volume restoration after 6 months was observed in SG, compared with 44% ± 5.5% in CG, as shown from MRI (Figs. 5B, 6B). This difference in terms of fat volume maintenance was statistically significant (*p* < 0.0001).

An average of 76% ± 5.0 and 69% ± 5.0% maintenance of fat volume, respectively, at T3 (1 month) and at T6 (after 6 months) (Fig. 5B) was observed in SG, compared with 52% ± 5.0 and 44% ± 5.5% in CG, respectively, at T3 and T6, as shown from MRI at T6 (Fig. 6B). This difference in terms of fat volume maintenance was statistically significant (*p* < 0.0001). At the same time, the maintenance rates in SG and CG were not similar in breast and face soft tissue defects. In fact, 70% ± 5.0 and 61% ± 5.0% fat volume maintenance, respectively, at T3 and at T6 was observed in SG for breast treatment, while 82% ± 5.0 (T3) and 77% ± 5.5% (T6) for face treatment (*p* = 0.1128 not statistically significant) compared to 46% ± 5.0 and 36% ± 5.0%, respectively, at T3 and at T6 in CG for breast treatment, and 58% ± 5.0 (T3) and 52% ± 5.0% (T6) for face treatment (*p* = 0.0082 this difference is considered to be very statistically significant).

US showed oily cysts in 55.5% of soft tissue analyzed on average at T3, and 46.0% at T6, while MRI detected oily cysts only in 4.8% and 3.2% at T3 and T6, respectively. At T3, and T6, the cytosteatonecrotic areas identified on both US and MRI were unchanged (5.5%) (Table 1).

### Histological Analysis

**Fig. 7:**
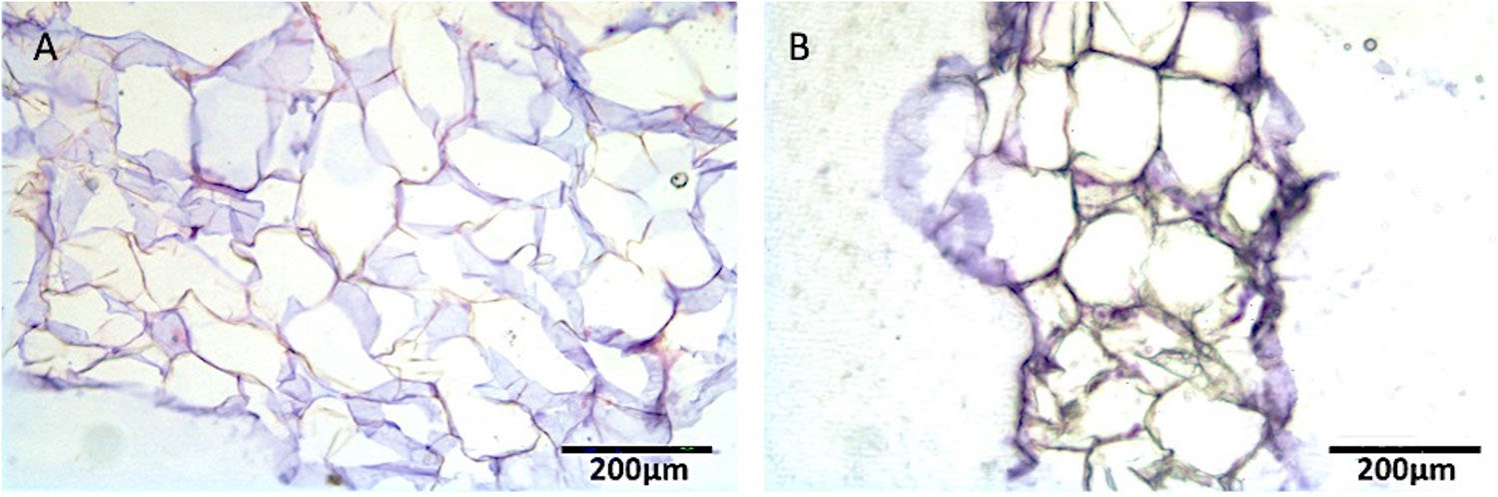
The micro-fragments obtained with both analyzed techniques were shown. Coleman fragments present sizes of about 1000 um, while Biopsybell generates smaller ones. Considering the histological analysis, the fragments obtained resulted in a well-defined and intact morphology for both cases

Figure 7A, B shows the micro-fragments obtained with both analyzed techniques, CG fragments present sizes of about 1000 µm, while SG generates smaller fragments. Considering the histological analysis, the fragments obtained resulted in a well-defined and intact morphology for both cases.

### Cellular Yield and Growth Capacity

**Scheme 2:**
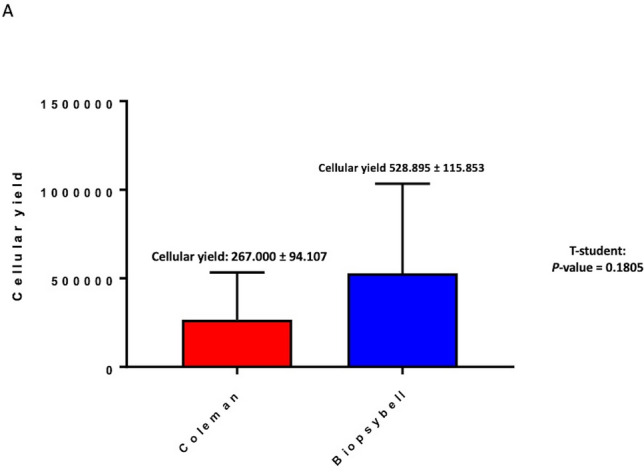
Cellular yield analysis of Biopsybell and Coleman extracted cells. (A) Graph representing the mean cellular yield of Coleman and Biopsybell. As shown, Coleman (control group) showed a cellular yield of 267,000 ± 94,107 ADSCs/mL, comparable to Biopsybell (study group) of 528,895 ± 115,853 ADSCs/mL, with no statistical differences. Data were analyzed with an unpaired t-student test. Quantitative data are expressed as means ± SEM. ns *p* > 0.05

Scheme 2A shows the results of both the cellular yield and cellular growth obtained with the total number of extracted ADSCs and the respective proliferative and growth capacity. In particular, the cellular yield of CG’s fat samples resulted in 267,000 ± 94,107 ADSCs/mL, while the cellular yield of SG’s fat samples was 528,895 ± 115,853 ADSCs/mL. The cellular yield analysis showed no significant differences between the SG and CG, with a *p*-value = 0.1805 obtained with a *t*-student test. This confirms that the cell vitality and the cell yield do not change but depend on the product-derived volume of treated adipose tissue.

**Scheme 3:**
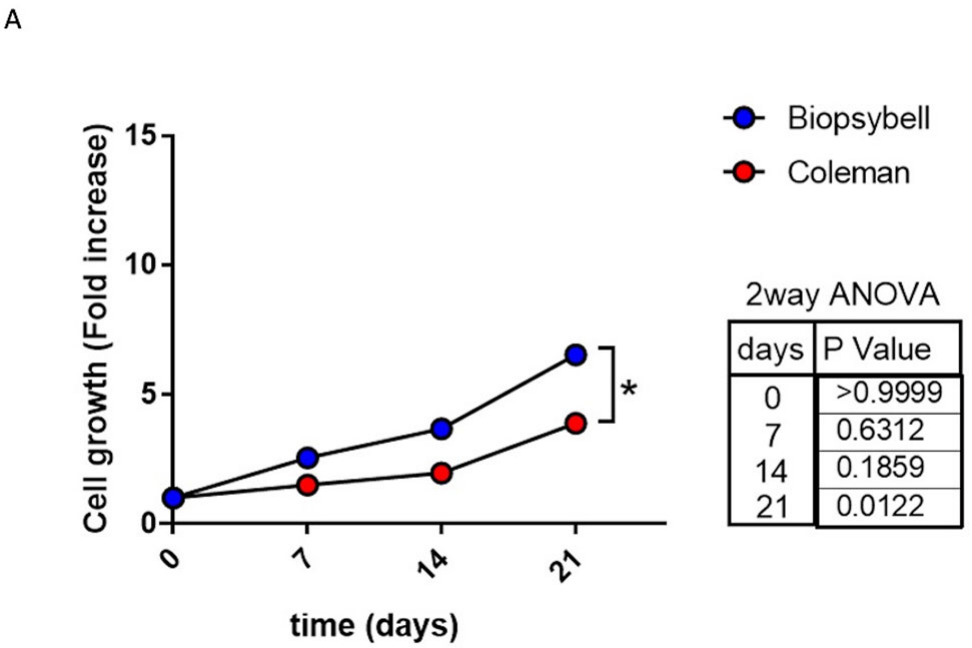
Cell growth analysis of Biopsybell and Coleman extracted cells. (A) Graph representing the cell growth expressed by fold increase of Coleman and Biopsybell. At 7 days and 14 days, Biopsybell cells (study group) showed a higher cell growth, even if no statistic, while 21 days resulted in significantly higher cell growth for Biopsybell (6758 ± 0.7122) if compared to Coleman (3888 ± 0.3078). Differences between experimental conditions were analyzed with a 2-way ANOVA test and post-hoc Tukey post-test. Quantitative data are expressed as means ± SEM: * *p* ≤ 0.05

On the other side, the cell growth resulted in higher statistical difference, for the stem cells obtained from the fat samples labeled as SG, if compared with the CG, with a *p*-value lower than 0.05 (*p*-value = 0.0122) obtained with the 2-way ANOVA test, at the last time point of analysis (Scheme 3A). In particular, the fat samples labeled as SG showed a fold increase in cell growth of 6758 ± 0.7122, while the samples of CG resulted in 3888 ± 0.3078. The improved cell growth ability was observed just starting from 7 days of culture, suggesting that the stimulated growth was influenced on the surgery day, depending on the technique.

### Strengths and Limitations of the Study

A main important strength characterized by this study was a detailed analysis of fat maintenance obtained from instrumental evaluation, based on MRI, US, and histological analysis.

On the other hand, several limitations characterized the presented work. Firstly, the study is preliminary, in fact regarding the timing, the analysis was limited to 6 months and not 12 months as usual. Secondly, the anatomical sites treated (breast, and face) were different. These differences made the groups heterogeneous and influenced the fat maintenance percentage as shown. Thirdly, for every patient, the amount of lipofilling to inject and the related amount of fat to collect were decided on the kind of defect and fat tissues available. Fourthly, the number of patients analyzed in SG and CG was limited (only 10 patients for each group).

## Discussion

The use of SVF in which ADSCs are contained is rapidly expanding in regenerative plastic surgery. As reported, the main method of fat digestion aiming to obtain SVF isolation is enzymatic, while the main strategies to obtain purified lipofilling are mechanical, each of them with several advantages and disadvantages [12].

Enzymatic digestion uses enzymes such as collagenase, trypsin, or dispase to dissociate the fatty tissue and release the mesenchymal stem cells. However, they are time-consuming, as they need an average of 60–90 minutes to digest the tissue and wash the enzymes out. Furthermore, enzymatic methods are more expensive than mechanical methods [12]. However, a great advantage of the enzymatic methods is the number of SVFs and ADSCs they yield. In conclusion, it could be argued that enzymatic methods are time-consuming, expensive, and require specific manipulations and knowledge [12]. On the contrary, mechanical processing allows the obtaining of purified lipofilling with a good amount of ADSCs within the SVF, with good viability, time-saving, and lower costs [12]. At the same time, mechanical procedures are based on different strategies (filtration, washing, fragmentation, centrifugation, etc.), some of them requiring special equipment and others being simple and cheap [12, 20, 21]. Most of them include some basic steps: washing, filtration, and centrifugation to concentrate the SVF and ADSCs into the purified fat [12, 20, 21]. The procedure described in the present paper manages to purify the lipofilling maintaining a good SVFs amount with excellent viability through simple and widely used means exclusively based on two procedures: washing and filtration. This procedure appears cheap, fast, and reliable, compared to other mechanical methods. The time needed to obtain lipofilling is short (ranging between 5 and 45 minutes, depending on the amount of injecting fat necessary), thus making mechanical methods ideal for one-stage procedures. The procedures are completely safe since they use autologous tissue, and the stem cells are not processed by any chemical or enzymatic means. Regarding mechanical processing, a recent study, showing the differences between mechanical and enzymatic procedures, in terms of SVFs amount and fat volume maintenance, was conducted by Gentile et al [12]. In this study, 80 patients affected by face and breast soft tissue defects were treated using different lipofilling procedures. In detail, 20 patients were treated using lipofilling enhanced with SVFs obtained by enzymatic digestion, 20 patients with SVFs obtained by centrifugation with filtration, 20 patients with SVFs obtained by only filtration, and 20 patients were treated with lipofilling obtained by only centrifugation according to the Coleman technique. In patients treated with lipofilling enhanced with SVFs obtained by automatic enzymatic digestion, a 63% ± 6.2% maintenance of fat volume restoration after 12 months was observed compared with 52% ± 4.6% using centrifugation with filtration, 39% ± 4.4% using only centrifugation (Coleman), and 60% ± 5.0% using only filtration [12]. Cell yield analysis showed that filtration was the most efficient system between mechanical digestion procedures thanks to the highest amount of SVFs obtained with fewer cell structure damage, producing the most volume maintenance after 12 months [12]. In the same line, in the present work, ADSCs seem to have an increased growth ability for the SVF obtained with the washing and filtration procedure, if compared to the standard Coleman technique based on centrifugation of 3000RPM (average 1100 *g*). The superiority of washing and filtration over centrifugation could be due to the mechanical issues of the *g* force, as suggested by Kurita et al [22]. Kurita et al. [22] investigated the effects of centrifugation at 400, 700, 1200, 3000, or 4200*g* for 3 minutes on liposuction aspirates reporting two important findings:

- centrifugation concentrated adipose tissues and ADSCs in the adipose portion and partly removed red blood cells from the adipose portion;
- centrifugation at more than 3000*g* significantly damaged ADSCs.

Therefore, excessive centrifugation can destroy adipocytes and ADSCs, but appropriate centrifugation concentrates them, resulting in enhanced fat graft maintenance. Kurita et al. [22] recommend 1200*g* as an optimized centrifugal force for obtaining good short- and long-term results in fat transplant.

Regarding the mechanical procedures based on washing, and purification of fat tissue, two different studies performed by Schafer et al. [23] and Zhu et al. [24] in which Puregraft^®^ was used, displayed augmented tissue viability, and reduced quantity of red blood cells, free lipids, and contaminants when compared to other lipofilling. Additionally, a study performed by Dos-Anjos Vilaboa et al. [25] displayed the results obtained by fat tissue washed and purified via GID 700™ displaying significantly reduced amounts of lactate dehydrogenase, triglycerides, and hematocrit maintaining the adipose graft osmolarity. SVFs obtained by the filtration device LipiVage™ displayed endothelial and mesenchymal progenitor cells, maintaining their differentiation capacity when used as fibrin spray [26]. Ferguson et al. [27] additionally reported that LipiVage™ produced a higher number of adipocytes and sustained a higher level of intracellular enzyme (glycerol-3-phosphatase dehydrogenase (G3PDH)) activity within fat grafts. A study performed by Bianchi et al. [28] reported that cells obtained from Lipogems^®^ showed a significantly higher concentration of mature pericytes, ADSCs, exosomes, and a lower quantity of hematopoietic cells when compared to isolated cells with enzymatic digestion. Condé-Green et al. [29] conducted a prospective cross-sectional study in which fat obtained by liposuction of the lower abdomen was separated and processed by decantation, washing, and centrifugation. Cell count per high-powered field of intact nucleated adipocytes was significantly greater in decanted lipoaspirates, whereas centrifuged samples showed a greater majority of altered adipocytes [29]. MSC concentration was significantly higher in washed lipoaspirates compared to decanted and centrifuged samples [29].

A very recent systematic review published in the current year 2023 by Langridge et al [30] confirmed the superiority of washing and filtration procedures. 24 studies (2413 patients) on fat processing techniques including centrifugation, decantation, washing, filtration, and gauze rolling, as well as ADSCs enrichment methods using commercial devices were identified and analyzed in this systematic review [30]. Complications were infrequent; palpable cysts (0–20%), surgical-site infections (0–8%), and fat necrosis (0–58.4%) were the most reported [30]. No significant differences in long-term volume maintenance between techniques were found in fat grafting of the breast. In head and neck patients, greater volume maintenance was documented in ADSCs enrichment (64.8–95%) and commercial devices (41.2%) compared to centrifugation (31.8–76% [30]). Langridge et al [30] concluded graft processing through washing and filtration, including when incorporated into commercial devices, results in superior long-term outcomes compared to centrifugation and decantation methods. ADSCs enrichment methods and commercial devices seem to have superior long-term volume maintenance in facial fat grafting [30].

As reported in the current work, and as analyzed from the above-mentioned studies a lack of a single standardized protocol both for the lipofilling purification and its enhancement with SVFs, must be considered as a limitation, as prevents having detailed predictability of the outcomes in terms of fat volume maintenance.

## Conclusions

Mechanical methods for purifying adipose are safe, cost-effective, and suitable for one-stage procedures, favoring comfort for patients. The method based on the washing and filtration procedure here described appeared effective, quick, safe, and can be easily reproduced, compared with centrifugation both in terms of ADSCs amount, viability, and fat volume maintenance. Additional perspective studies and controlled trials will be necessary to confirm the preliminary data here reported.

### Supplementary Information

Below is the link to the electronic supplementary material.

Supplementary file1 (JPEG 397 KB)
