# Supplementary figures and images for: Mechanical Purification of Lipofilling: The Relationship Between Cell Yield, Cell Growth, and Fat Volume Maintenance
Source: Aesthetic Plast Surg. 2024 Mar 20;48(12):2306–18. doi: 10.1007/s00266-024-03870-0 (PMC11233364; doi:10.1007/s00266-024-03870-0)

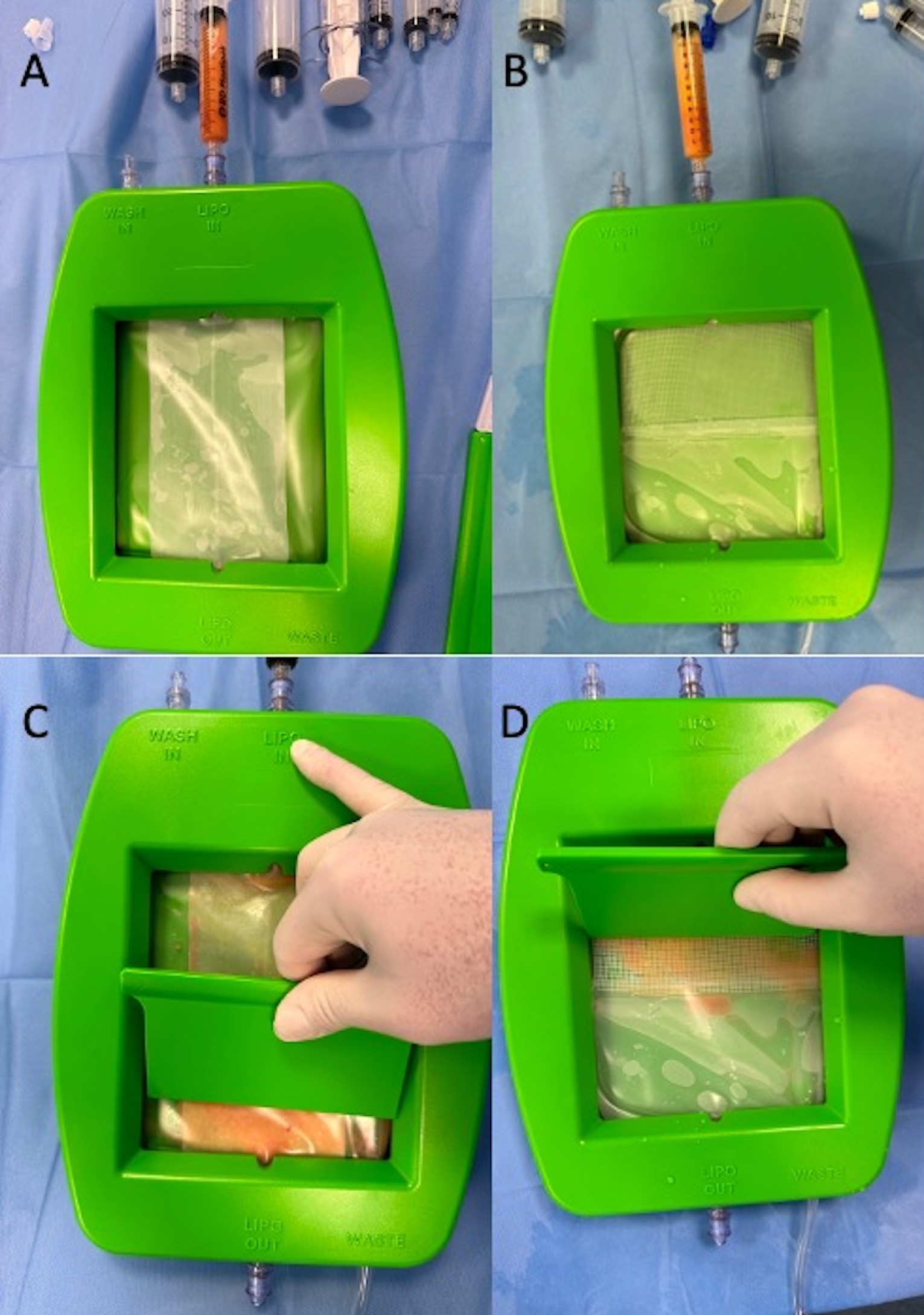

Supplement: Supplementary file 1 — Supplementary file1 (JPEG 397 KB) [file 266_2024_3870_MOESM1_ESM.jpeg]
